# Find your village: a call for culturally coordinated understanding and action with migrant heritage families in the Global North

**DOI:** 10.1080/16549716.2026.2732764

**Published:** 2026-10-07

**Authors:** Tom Allport, Fatumo Osman

**Affiliations:** a Centre for Academic Child Health, University of Bristol Medical School, Bristol, UK; b Community Children’s Health Partnership, Sirona Care & Health, Bristol, UK; c School of Health and Welfare, Dalarna University, Falun, Sweden

**Keywords:** Migrant, child development, social support, parenting, cultural competence

## Abstract

Refugees and forced migrants coming to the ‘Global North’ experience high rates of stress and isolation. Migrant communities are concerned about their young peoples’ long-term health, educational and social outcomes; there are reports of their children being referred at higher rates with early developmental difficulties and subsequently learning difficulties and conditions such as autism. We argue that these distressing experiences are interconnected and understandable in context, and we would like to engage with these accounts as indicating a need for more understanding, debate, negotiation, opportunities, connections and resources. Parents from cultures where ‘it takes a village to raise a child’ may find it particularly challenging to bring up children in individualistic western societies when they also experience stress, trauma, isolation and environmental disadvantage. In a linked paper, we describe an Ecocultural model that seeks to explain challenges to parental wellbeing and early child development for Somali refugee families in its local and international context, and the development of a flexible approach to targeted, preventive peer support intervention we have called Find Your Village, which has the potential to meet these challenges. In this debate paper, we advocate for the development of culturally coordinated understanding and action, describing three ‘pillars’ to build a ‘village’ for migrant children and families in the Global North, and suggest resources and approaches that have the potential to stop cycles of disadvantage, provide multiple benefits, and save costs to society through co-production, inequality reduction and integration.

## Paper Context

- **Main findings:** The challenges for refugees and forced migrants from cultures where ‘it takes a village to raise a child’ for bringing up children in the Global North need understanding and addressing.
- **Added knowledge:** We advocate for the development of culturally coordinated understanding and action by services and communities, describing three ‘pillars’ to build a ‘village’ for migrant children and families in the Global North, and suggest resources and approaches that have the potential to stop cycles of disadvantage, including a flexible approach to targeted, preventive peer support intervention that we have called Find Your Village.
- **Global health impact for policy and action:** We anticipate that implementing such approaches sustainably would have the potential to improve life-chances, benefit families and communities, reduce a range of adverse outcomes for health, wellbeing, challenging behaviour, education, employment and crime, save money for services, and inform national policy and practice.

## Background

For refugees and forced migrants, the journey to a country in the ‘Global North’ brings many challenges. Arriving in a very different society brings many further challenges – often insecure, impoverished environments, limited resources, a new language, discrimination/racism, and (perhaps most powerfully) individualisation and isolation [1–3]. Negotiating parenthood and maternity care in a new country brings yet further demands and stresses [4,5]. These complex, interacting challenges affect parenting and parent–child relationships, often bringing feelings of disempowerment and a lack of self-efficacy. Seeing each one of us as having a ‘backpack’ that contains the experiences that we carry with us, the challenges or disasters we have survived [6], the people we have lost along the way, as well as the positive experiences, values, practices and needs we bring with us and which services need to be able to respond to (which can be summarised as choice, trust, empowerment, collaboration and safety; www.traumatransformation.scot). In this paper, we seek to direct the attention of health care and other professionals in the ‘Global North’ towards support for refugee and forced migrant families navigating their new context.

From our observations over many years as practitioners and researchers (the co-authors have a combined experience of over 45 years of working with local Somali communities in the Global North), we argue that the suffering of migrant parents, and their children’s distress and early developmental difficulties, may be interconnected and understandable in contexts of stress, trauma, isolation and environmental disadvantage. We use the term ‘environment’ here to signify the social (people, settings and activities) and physical (housing, communal space and outdoor built and natural) environments within which families meet, parents receive support and children play, in the understanding of multiple cumulative exposures with reciprocal effects influencing child development across the lifecourse [7,8]. Our research places particular significance on the transition from communal or collective cultures where ‘it takes a village to raise a child’ to more individualist environments. We believe this represents an important opportunity for tailored early interventions and also may offer important lessons for host populations. This prompts a series of interrelated questions. What stories, concepts and actions may be relevant to refugees and forced migrants bringing up children [9]? What can help them establish themselves and raise children in their new country? What do their children need in order to thrive? How can we help co-create the sense of ‘village’ that families need around them, to experience connection, wellbeing and support?

## Context

By the term ‘Global North’, we do not seek to refer to a geographic region in any traditional sense but rather to the relative power and wealth of countries in distinct parts of the world, while acknowledging that not everyone in the ‘Global North’ is rich and powerful [10]. Inrelation to a ‘Global South’ construct within epistemological, political, and historical processes that addresses spaces and peoples negatively impacted by globalisation, while seeking to identify the social agency of dominated groups [11,12]. While only a relatively small proportion of refugees and forced migrants are hosted by countries in the ‘Global North’, academic research on forced migration is predominantly published by ‘Global North’-based institutions; most research methodologies and metrics are deeply rooted in western, state-centric perspectives rather than the lived experiences of refugees and forced migrants themselves [13,14]. Acknowledging these multi-layered inequalities and injustices, this Debate paper attempts to bring some of the discomfort and dislocation of human experiences into a frame of community-agency relationships, as part of the financial, psycho-social and environmental complexity of a move from ‘Global South’ to ‘North’ [3,15].

Our review of the systematic reviews and other evidence about the needs of refugees and forced migrants to the ‘Global North’ focuses on the need for interventions addressing loneliness and isolation [3,16,17], parenting [18–22], and focusing on maternity and post-natal periods [23–27], while attending to cultural adaptation, recruitment and scalability. There are also pressing concerns about a broad range of outcomes for children and young people from a disadvantaged migrant background, including mental health difficulties, poor educational and employment outcomes, crime and potential for radicalization [28,29]. There have been reports viewing children of refugees and forced migrants in the ‘Global North’ at higher risk of delayed and disordered early development, and subsequently learning difficulties and conditions such as autism [30–32], amongst discussion of possible environmental factors for autism including those contributing to stress in pregnancy [33,34]. We would like to engage with these accounts as indicating a need for more understanding, debate, negotiation, opportunities, connections and resources, looking ‘beyond blame’ to inclusive and interconnecting action.

Inequalities in pregnancy and early childhood have powerful and lifelong effects on children, affecting developmental and mental health outcomes as well as life chances, drawing increasing attention and priority towards effective, early intervention [35–41]. Young children need opportunities for play and social interaction to develop and socialise confidently. Longitudinal research highlights the importance for later educational outcomes of high‑quality, play-based early learning experiences [42,43]. Interventions to improve early child development and support parenting are highly effective and cost-effective for a wide range of youth and adult outcomes [44–47]; however, multiple intersectional factors including ethnicity & culture challenge engagement with and experience of routinely offered antenatal, early childhood and parenting services by [48–51]. In our experience, services assume they can apply the same ‘offer’ that is provided for the host population, without gathering or critically reviewing data about accessibility, uptake or relevance. Culturally relevant interventions may be adopted on a ‘project’ basis but funding is not sustained and the approach not embedded into wider service provision.

We believe the resource limitations, inequalities and marginalising processes experienced by many migrant communities are highly significant for their outcomes. Pointing to the inadequacy, inequality, and unsustainability of the resourcing of their communities and environments may help to challenge narratives of deficit or failure often used unwittingly by governments and service providers that serve to blame families or ‘reinscribe’ them with the effects of the structural inequalities they experience [52]. Commissioners requiring measurable ‘data’ before instituting change when current service provision is inaccessible, inappropriate or untrusted would appear to serve as a potent mechanism for perpetuating or exacerbating inequalities.

This Debate paper responds to these concerns, the lived experience of the many families we have met, and the findings of our own and others’ research, to call for interventions to be developed from shared local and cultural knowledge and implemented sustainably.

## Local, cultural understanding and action for migrants to the ‘Global North’

In a linked research paper [53], we report the construction of an ecocultural model of predisposing factors appearing to challenge parent wellbeing and early child development in Somali migrants to the ‘Global North’, and from this describe the development of a flexible approach to peer support intervention co-design, transferable to local contexts. This model describes challenges that occur at the meeting point (through family migration) between contrasting African/Somali and Western/European approaches to child-rearing [2,3,9,54,55]. It may be especially challenging for parents who themselves grew up in cultures where ‘it takes a village to raise a child’ to bring up children in the Global North, with multiple disadvantages and marginalising processes, and away from supportive social networks [3,56,57].

## Asset-based approaches to intervention and change

In these complex contexts, the social support and structure available within communal cultures represent latent assets with great potential (see short film at https://vimeo.com/374652881/79fae10d7e). Strong communities build wellbeing, security and social connectedness, which are likely to be especially important for families from ‘communal’ or ‘collective’ child-rearing cultures, and play critical roles in child and youth outcomes. We need to advocate for and co-create environments that promote secure infant-parent attachments and confident child-rearing, rather than those that generate chaos, isolation and trauma [58]. In our linked paper, we describe potentially modifiable factors that could be addressed, and key processes that, combined, can achieve this in peer support interventions tailored to local contexts. While this approach has been developed primarily with Somali partners, our broad network of community and statutory partners indicates such an approach appears applicable to parents from communal/collective cultures and societies where child-rearing may be communal/collective [9]. We note that in the most recent statistics available, 22-24% of births in England and Wales had one or both parents were born outside the ‘Global North’ [59]; 37.5 million EU residents were born beyond the Union’s borders (8.4% of all EU inhabitants); the Somali diaspora is estimated at 2 million people [60]. The concept of ‘village’ may also have relevance to supporting social inclusion for other disadvantaged families. We are therefore seeking to build national and international networks to support such models and approaches being adopted more widely, and embedded sustainably within service provision (e.g. https://ladnaan.se/home/).

## Culturally coordinated interventions and services

How can the interventions that agencies and services develop and implement be culturally coordinated with families’ needs? Trust can be a key factor, to the extent that it can be considered a key social determinant of health [59]. Professionals working with migrant families therefore need to have cultural competence and knowledge [60] to address stressors, build on strengths, and counter expectations, following experiences of discrimination and racism, that professionals will judge them negatively and offer inappropriate or lesser services [2].

## Three pillars to build a ‘village’

To bring these thoughts together, we describe three pillars to build a ‘village’ for migrant children and families in the Global North (Figure 1):

**Figure 1.**
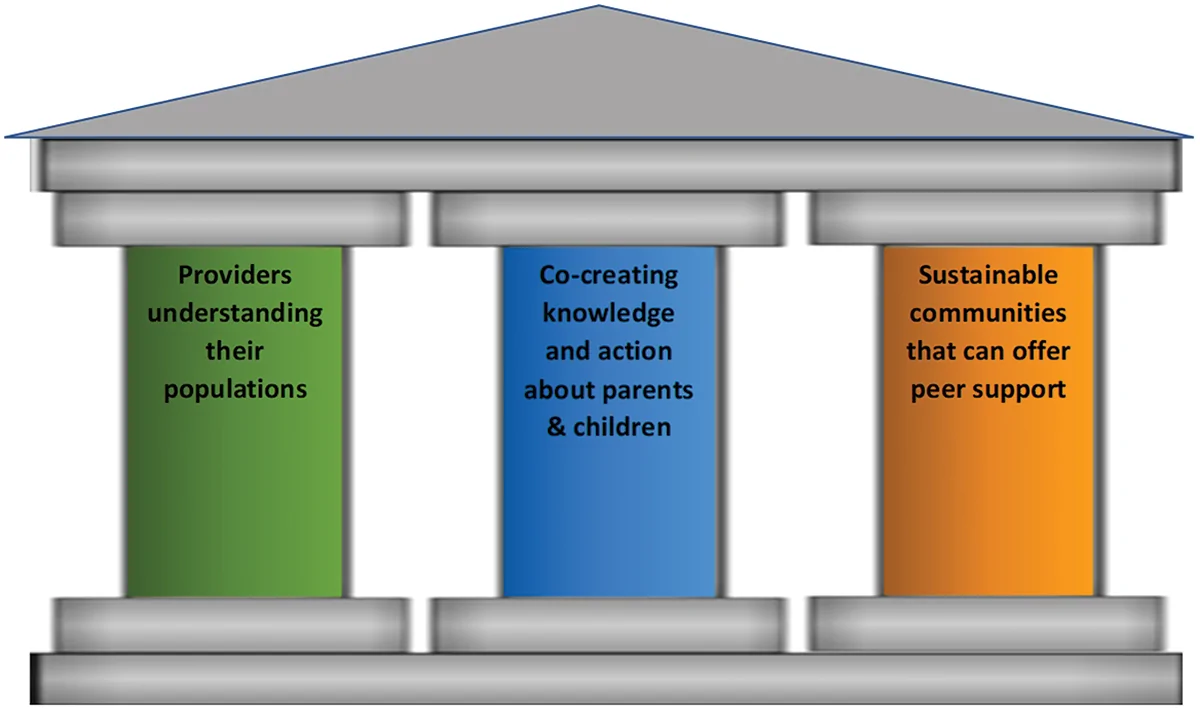
Three pillars for building a ‘village’ for migrant children and families in the Global North.

- Healthcare and other providers understanding the populations they work with;
- Sustainable communities that can offer peer support and help parents in turn feel able to contribute to their society; and
- Providers and communities co-creating knowledge and action about children and families, to improve relationships and outcomes and reduce inequalities.

Before describing the three pillars, we first introduce a vignette, as an illustrative case-example (not a real family) which we will then use to illustrate each pillar.

## Vignette: navigating resettlement and health

Mona fled the civil war in her home country with her husband and their two children. They arrived in the UK as asylum seekers and waited 3 years before receiving residence permits. In their home country, both Mona and her husband had worked for years as teachers. However, in the UK, they were unable to find employment in their profession, and the family struggled financially during their early years of resettlement. The transition was difficult. Without stable employment and with limited familiarity with the system, the family faced ongoing challenges in meeting their daily needs.

Mona is pregnant and attends regular check-ups with her midwife. Recently, she has also had several appointments with her GP, seeking support because she has not been feeling well. At each visit with both the GP and the midwife, she was told that she did not have any specific health conditions but was advised to lose weight, eat a balanced diet, and avoid foods such as rice and bread, as these were assumed to be common staples in her culture. The midwife additionally advised Mona not to eat too much.

At the same time, the school nurse contacted Mona and her husband to inform them that two of their children, Afrah and Isa, were overweight. The nurse suggested that the parents reflect on the types of food they were providing at home. She also expressed concern about the children’s overall development and their social interaction, noting that they do not play with their peers at school.

The family had no extended social network in the UK. Outside of school and occasional obligations, they spent most of their time at home. This isolation, combined with financial constraints and limited access to culturally appropriate support, shaped the family’s daily routines, including their eating habits and opportunities for physical activity.

## Providers understanding their populations

Our previous work describes migrant parents’ wish to have providers who understand them – both their history and their current strengths and needs. This therefore includes what they have in their ‘backpack’ (their experiences before, during and since migration), their love for their children, the individual and family challenges they are going through, and their desire for connection, for a sense of village in their new context [2,9].

This first pillar depends on cultural sensitivity – providers do not necessarily need to know families’ culture in advance, but rather not to come from biased, judgmental standpoints. Instead, they need to be open to developing the understandings together that are needed, to working relationally [61,62], to ‘*thinking with*’ [63]. Roles, duties and responsibilities of different service providers will differ in each country and local area, therefore we have tried not to detail these as specific descriptions would then risk not applying to other countries. Attention to what builds trust can be the key to effectiveness [64–67].

The vignette illustrates the healthcare and school systems’ lack of understanding of the family’s context. The advice given focused narrowly on weight and diet, without considering the family’s financial constraints, social isolation, or desire to rebuild a sense of community and belonging. Understanding these dimensions could have enabled more relevant and supportive care.

## Sustainable communities that can offer peer support

The second pillar requires communities being sustainably resourced, and therefore able to offer mutual peer support. Families have so much to offer that, supported and strengthened, can benefit both themselves and the wider community. However, multiple interconnected economic, social and environmental barriers prevent them from sharing these assets and from health interventions working effectively and equitably [68,69].

Alongside the need for broad structural change to reduce barriers, inequalities and injustices, we argue that community and other civil society organizations have a crucial role to establish and enable sustainable peer support networks [65,66]. From a longer-term perspective, it is crucial that services collaborate with community organisations to strengthen their capacity to support families in similar situations. Such partnerships can begin to build inclusive, accessible support systems that address both social and health needs.

The vignette illustrates how, while Mona and her family have much to offer their community, structural barriers such as social isolation and limited access to local networks prevent these strengths from being realised and from supporting their health and wellbeing. Therefore, it is important to support the family by involving and connecting them with social and community-based activities and centres.

## Co-creating knowledge and action to support parent–child relationships

Our third pillar is the meeting point between the first two. Providers need to acknowledge parents’ and families’ existing knowledge and actions, build upon these, celebrate successes and help them recognise what else may be needed.

In the vignette, such a meeting point is largely absent. As parents with professional backgrounds as teachers, they bring valuable skills and insights, yet they are primarily given directive advice rather than being engaged in reflective dialogue. This highlights a missed opportunity for providers to support a process of co-creation – where parents’ strengths and needs can be recognised and responded to, most valuably from early in their and their children’s journeys [70].

In another example from our previous work, parents we have interviewed recognised an authoritarian parenting style that had worked in their home country was now not being effective. Their children’s opposition to this approach helped them to realise the need for change [71]. At this point of readiness for change, providers bringing evidence-based parenting programmes that encourage self-reflection can co-create with parents changes in parenting practices that strengthen relationships with their children and improve outcomes [18,22]. At the same time, communities need to develop their place as ‘critical friends’ to work with, challenge, and guide policy, service provision and accessibility [72], which in turn depends on their emancipation, organisation, and resourcing.

## Conclusions

We have articulated a need for culturally coordinated understanding and action by services and communities, describing three ‘pillars’ to build a ‘village’ for migrant children and families in the Global North. Local examples can help to articulate the challenges, needs and solutions that can be built on ‘at scale’ [73]. A flexible approach to targeted, preventive peer support intervention that we have called Find Your Village [53] has the potential to meet many of these challenges. Implementing such interventions, and the community resourcing and cultural coordination to underpin their success and sustainability, would require the following resources: staff training and time to tailor and target statutory interventions; broad organisational commitment to respectful, power-sharing relationships that build truly co-created understanding and action; and concerted, sustained intersectoral investment to reduce inequalities in the built and social environment so that diverse communities can thrive. We anticipate such approaches would have the potential to stop cycles of disadvantage, improve life-chances, benefit families and communities, reduce a range of adverse outcomes for health, wellbeing, challenging behaviour, education, employment and crime, save money for services, and inform national policy and practice. We hope this Debate paper can assist the broad audience of Global Health Action to adopt and adapt the ideas and principles presented here to their own contexts.

The increasing consensus in public health that achieving inequality-reducing system-based change in underserved communities experiencing structural inequalities requires co-production or co-creation [74–78], together with increasing attention to neighbourhood-based and integrated commissioning of health and other services [79–83], represent promising opportunities to move things forward. Concerted global heath action in this area seems much needed.

## Data Availability

There is no specific dataset linked to this article, but further information can be made available by the corresponding author on reasonable request.
